# Contrast-Induced Sialadenitis of the Sublingual Glands

**DOI:** 10.1155/2020/8851382

**Published:** 2020-09-08

**Authors:** Ki Wan Park, Albert Y. Han, Christine M. Kim, Kenric Tam, Dinesh K. Chhetri

**Affiliations:** ^1^Department of Otolaryngology-Head & Neck Surgery, Stanford University, Stanford, California, USA; ^2^Department of Head and Neck Surgery, University of California, Los Angeles, California, USA

## Abstract

Contrast-induced sialadenitis (CIS) is a rare, delayed pseudoallergic reaction from iodine containing contrast. Previously reported cases of CIS demonstrated that the two major salivary glands (parotid and submandibular) can be affected. The initial encounter of this entity can raise alarms to physicians as the differential diagnoses include serious infectious and inflammatory conditions such as Ludwig's angina and angioedema. Subsequently, it may lead to unnecessary testing and increased healthcare cost. Here we present a 60-year-old male who presented with bilateral sublingual gland swelling following exposure to iodinated contrast. With timely diagnosis by the otolaryngologist, the patient received conservative management that led to a full resolution within a few days. To date, this is the first case of CIS only involving the sublingual glands. We conclude that CIS can involve any of the major salivary glands.

## 1. Introduction

Contrast-induced sialadenitis (CIS), also known as iodide mumps, is a delayed side effect observed after exposure to iodine-containing contrast. Most commonly, CIS involves painless swelling of the salivary glands. The first case report was published in 1956, with a patient who developed contrast-induced sialadenitis or “iodide mumps” after intravenous urography [1]. Similar inflammatory reactions have also been reported in the pancreas [2]. One study assessing reactions to contrast material in 1,381 patients found a CIS incidence rate of 1-2% [3]. Given its benign course, prompt diagnosis is essential to avoid unnecessary workup including computed tomography (CT) scans, magnetic resonance imaging (MRI) scans, and blood tests. Furthermore, all previously documented cases of CIS in the literature have involved either the submandibular or parotid glands [4]. Here we present a case of CIS involving the sublingual glands and review the current literature on the pathogenesis, clinical presentation, and treatment.

## 2. Case Presentation

A 60-year-old male with a history of hemochromatosis status postorthotopic liver transplant (on immunosuppression), type 2 diabetes, chronic kidney disease (baseline creatinine 1.4), and prior right common femoral deep venous thrombosis presented with non-ST-elevation myocardial infarction (NSTEMI) at an outside institution. The patient underwent coronary angiography at the outside institution and was placed on dual antiplatelet therapy. No information regarding contrast type and premedications prior to coronary angiography was available. Given liver transplant status and persistent elevations in troponin, the patient was transferred to our institution for a higher level of care. One day after coronary angiography at the outside institution, the patient underwent repeat angiography and percutaneous coronary intervention (PCI) at our institution. No premedications were administered prior to the procedure. The patient had no history of any allergies and had a baseline Cr of 1.4. Angiogram was performed with 165 ml of iodixanol 320 mg/ml (Visipaque), an iodine-containing nonionic radiocontrast agent, over a 60 min interval. The procedure was performed under conscious sedation without any manipulation of the mouth or throat. Four hours following the procedure, the patient presented with painless swelling of the floor of mouth (FOM), and otolaryngology was consulted. At the time, the patient also denied any dyspnea, dysphonia, or neck pain.

**Figure 1:**
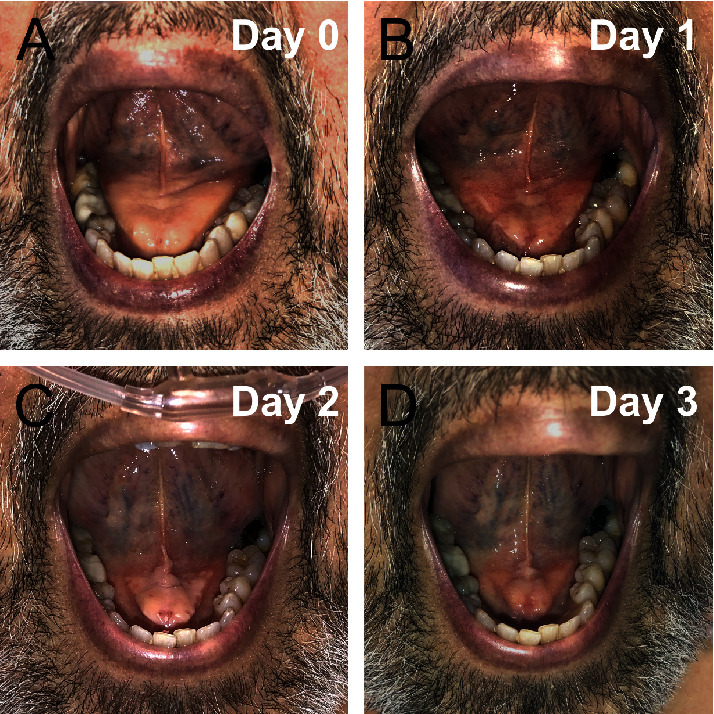
Clinical course of the patient with contrast-induced sialadenitis. (a). Day 0 (b). Day 1 (c). Day 2 (d). Day 3.

On examination, the patient was afebrile, with a pulse of 93 beats/min, blood pressure of 106/69 mm/Hg, and a respiratory rate of 20 breaths per minute. Painless swelling of the FOM consistent with swollen sublingual glands was present without any evidence of mucopurulent drainage or fluctuance (Figure 1(a)). The rest of the physical exam was unremarkable.

Differential diagnosis included allergic etiologies (angioedema), sialadenitis, acute infection (e.g. Ludwig's), and contrast-induced sialadenitis. Given history of recent contrast exposure, localized bilateral swelling, and no acute signs of infection, patient's presentation was most consistent with CIS involving the sublingual glands. The patient was administered with diphenhydramine perorally for treatment.

**Figure 2:**
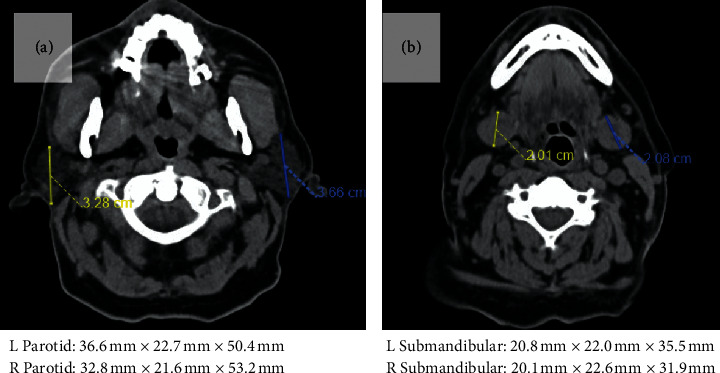
CT neck w/o contrast axial views demonstrating no enlargement of either the parotid gland (a) or submandibular gland (b).

Overnight, the patient became febrile and septic with increasing oxygen requirements likely secondary to cardiac etiology versus pneumonia. Physical exam revealed decreased swelling of the sublingual glands. Blood cultures and labs were remarkable for elevated creatinine (2.4) consistent with acute kidney injury (AKI). Etiology of shock remained unclear but was thought to be an infectious source. Broad spectrum antibiotics were prophylactically started on the patient given immunosuppressed status. The CT of the neck and chest without contrast was also obtained by the primary team to rule out any occult deep neck space infection given immunosuppressed status. CT neck and chest was unremarkable, with no evidence of masses, infectious processes, or airway compromise. No enlargement of either the parotids or the submandibular gland was noted on imaging (Figures 2(a) and 2(b)). While no sublingual swelling was visualized, the CT was performed 36 hours after contrast insult and without contrast. Subsequent physical exams revealed decreased swelling until full resolution at 3 days (Figures 1(b) and 1(d)).

## 3. Discussion

CIS has been increasingly reported in the literature since the first case report in 1956. To date, over 70 cases have been reported worldwide in the literature. There is no predilection for age or gender, with age ranging from 8 to 83 years [4]. The submandibular gland is the most common affected salivary gland (37/77 cases) followed by the parotid gland (21/77 cases), with many cases involving both glands (19/77 cases) [4]. To our knowledge, this is the first documented case of sublingual gland involvement following iodinated contrast exposure.

The pathophysiology of CIS has yet to be delineated but is thought to be a pseudoallergic reaction due to accumulation of iodine in the glands. After intravenous injection, 98% of iodine is excreted renally with the remaining 2% concentrated in sweat, parotid, and minor salivary glands through the sodium iodide symporter [5]. It has been studied that iodine concentrations in the glands can exceed 100x than that of the plasma [6]. This excess accumulation is thought to result in mucosal edema, obstruction, and subsequently sialadenitis [7]. Further studies, however, have also demonstrated that nonionic contrast can result in endothelial cell damage and vascular disruptions through changes in Ca^2+^ handling with stimulation of the Na^+^-K^+^ ATPase pump and altered Na^+^-Ca^2+^ exchange [5]. The time of onset for CIS after contrast exposure has a mean of 16 hours, but varies significantly between 0.1 hours to 120 hours [4].

Traditionally, it has been hypothesized that patients with renal impairment have been thought to be at a higher risk for CIS. Our patient who presented with sublingual swelling also had CKD and subsequently developed an AKI in the context of sepsis. However, numerous cases have reported CIS in patients without any renal impairment [8]. A recent meta-analysis by Jiao et al. assessing 69 cases of CIS found no statistical difference between patients with and without renal impairment [4]. Further studies are necessary in order to delineate this factor in the future.

Diagnosis of contrast-induced sialadenitis is clinical but can be aided with imaging. Ultrasound (US) findings typically demonstrate significant swelling of salivary glands with hypoechoic septa, increased vascularity, and dilated ducts without any evidence of sialolithiasis or infection [9]. CT and MRI have been shown to demonstrate nonspecific gland enlargement and edema. In our patient, the CT neck and chest did not reveal any airway narrowing or edema.

The clinical course of CIS is benign and often self-limited. No life-threatening sequelae have been reported in the literature to date. Thus, treatment is usually supportive with antistatistically significant predictors [4]. Interestingly, the contrast type and renal impairment were not predictors for resolution [4]. Our patient had full resolution of symptoms at 3 days following exposure to contrast and received only diphenhydramine for treatment. While our patient did not receive any additional iodinated contrast exposure, repeated iodinated contrast exposure is generally not recommended as recurrent sialadenitis is possible but may be permitted for life saving interventions [10].

To date, no uniform treatment protocol currently exists in the literature. However, as CIS is a benign condition, if necessary, most patients can be reasonably being managed with supportive measures including antihistamines, analgesics, and steroids. For refractory cases, more aggressive measures such as intravenous steroids can be considered.

## 4. Conclusion

CIS remains a benign, uncommon reaction to iodinated contrast used in procedures. The clinical course is self-limiting with full resolution within 14 days. Steroids and antiinflammatory agents have been used to expedite resolution. Here we provide the first documented case of contrast-induced sublingual sialadenitis. CIS can present in either sole or combinations of all three major salivary glands.
